# The Impact of Exercise in Rodent Models of Chronic Pain

**DOI:** 10.1007/s11914-018-0461-9

**Published:** 2018-06-23

**Authors:** Mark Henry Pitcher

**Affiliations:** Pain and Integrative Neuroscience Laboratory, National Center for Complementary and Integrative Health, National Institutes of Health, Room 1E-420, 35A Convent Drive, Bethesda, MD 20892 USA

**Keywords:** Chronic pain, Exercise, Rodent, Treadmill, Voluntary, Stress

## Abstract

**Purpose of Review:**

Physical activity is increasingly recommended for chronic pain. In this review, we briefly survey recent, high-quality meta-analyses on the effects of exercise in human chronic pain populations, followed by a critical discussion of the rodent literature.

**Recent Findings:**

Most meta-analytical studies on the effects of exercise in human chronic pain populations describe moderate improvements in various types of chronic pain, despite substantial variability in the outcomes reported in the primary literature. The most consistent findings suggest that while greater adherence to exercise programs produces better outcomes, there is minimal support for the superiority of one type of exercise over another. The rodent literature similarly suggests that while regular exercise reduces hypersensitivity in rodent models of chronic pain, exercise benefits do not appear to relate to either the type of injury or any particular facet of the exercise paradigm. Potential factors underlying these results are discussed, including the putative involvement of stress-induced analgesic effects associated with certain types of exercise paradigms.

**Summary:**

Exercise research using rodent models of chronic pain would benefit from increased attention to the role of stress in exercise-induced analgesia, as well as the incorporation of more clinically relevant exercise paradigms.

## Introduction

Chronic pain represents an urgent global health problem [1] that incurs massive social and economic costs [2, 3]. Highly prevalent, chronic pain affects between 19 and 43% of the US population [4–8]. A substantial portion of the chronic pain population is comprised of those with bone/joint pain [7], where osteoarthritis (OA) and rheumatoid arthritis (RA) are considered to be among the most disabling of the chronic bone/joint diseases [9–11]. At the population level, over 20% of adults under 65 years old, and almost 50% of adults over 65, have some form of arthritis [12]. A substantial proportion of this population have restricted joint motion, muscle weakness, substantial activity limitations, and are physically inactive [12–19]. As such, arthritis is thought to be the main cause of disability in the USA, with a socio-economic impact approaching $200 billion annually as of 2007 [20].

Three main treatment modalities are available for bone/joint diseases such as OA and RA: surgical, pharmacological, and non-pharmacological [21, 22]. While surgical and pharmacological treatments can certainly be beneficial [23, 24], these approaches are not without risk and/or unpleasant secondary effects [22, 25, 26]. Indeed, referrals for orthopedic surgical interventions are often indicated only after less invasive treatment options have been exhausted [27]. As such, non-pharmacological approaches are increasingly recommended as first-line treatments for certain types of chronic pain, even prior to pharmacological interventions [28]. An increasingly popular non-pharmacological approach is exercise, which has been defined as “planned, structured, and repetitive bodily movements that are performed to improve or maintain one or more components of physical fitness” [29]. The beneficial effects of exercise are undeniable, both for maintaining health and for reducing the negative impacts of many chronic illnesses including cancer, type 2 diabetes, obesity, and depression [30–33]. In this brief review, our goal is to survey the current state of knowledge on the effects of exercise on chronic pain outcomes both in humans and in rodent models of chronic pain. Considering the number of high-quality reviews and meta-analyses that have critically appraised the literature on the effects of aerobic exercise in chronic bone/joint pain in humans, we will only outline their main conclusions. We will, however, discuss the limited rodent literature in more detail. Finally, we will discuss these rodent findings in light of the human studies.

## Is Exercise Beneficial for Chronic Pain in Humans?

Habitual physical inactivity is one of the leading risk factors driving non-communicable diseases and death worldwide [34–37]. Sedentary lifestyles are associated with poorer health as well as reduced day-to-day functioning and quality-of-life [13, 38–40], where even intermittent bouts of vigorous activity appear to be unable to counteract the impacts of habitual physical inactivity [13, 39, 40]. Regular physical activity, however, can be effective in both prevention and treatment of many chronic diseases [30–32]. In healthy individuals, there is a long history of support for analgesic effects of regular physical activity, particularly highly aerobic exercises such as endurance running/cycling [41–43]. Additional benefits include the reduction of depression, anxiety, and stress [44–48]. Increasingly, guidelines state that exercise should be part of the core treatment for chronic pain [28, 49–52], generally advising low-to-moderate levels of physical activity that are increased incrementally (i.e. “*Start low, go slow*”) and monitored by a qualified health care professional. The effects of regular exercise for chronic pain and its comorbidities has been the subject of many primary research reports, reviews, and meta-analyses over the last 30 years. Rather than attempt to re-evaluate the surfeit of primary literature, we will instead briefly report the main conclusions from a few recent Cochrane reviews that have carefully collated this massive and diverse literature into a more digestible format.

Among the most up-to-date and comprehensive reviews is a Cochrane review from Geneen et al. [53••]. Geneen et al. assessed 21 previous Cochrane reviews, incorporating 264 primary reports and 19,642 participants, to determine the effectiveness of different physical exercise interventions in reducing pain stemming from various chronic pain syndromes. Overall, while exercise resulted in reduced pain severity and improved physical function in various forms of chronic pain, effects were small-to-moderate at best and were quite inconsistent across reviews. Exercise-induced effects on psychological function and quality of life were equally variable. Nonetheless, exercise was associated with few adverse events as well as improved pain severity, physical function, and quality of life. Similarly, in a 2014 Cochrane review assessing therapeutic exercise for hip OA in 9 trials (549 participants), Fransen et al. [54] reported high-quality evidence in support of exercise-induced improvements in pain and physical function in individuals with hip OA. In another Cochrane review from the same group based on 44 trials (3537 participants), Fransen et al. [55] reported that therapeutic exercise produced short-term improvements in pain and physical function in individuals with knee OA. However, the lack of blinding in most trials (i.e., participants being aware of their treatment group) may have contributed to the improvement.

In terms of the relative effects of different types or intensities of exercise, O’Connor et al. [56] assessed 17 studies on walking exercise in individuals with chronic low back pain, OA, or fibromyalgia. Overall, walking produced a small-to-medium improvement in pain and physical function in the short term, whereas longer-term effectiveness was uncertain. Regneaux et al. [57] included 6 reports comparing the effects of high- or low-intensity exercise in 656 participants experiencing hip or knee OA. Overall, high-intensity exercise did not seem to provide any clinical benefit over low-intensity exercise in terms of pain and physical function. Regneaux et al. [57] indicated that the paucity of studies comparing high- and low-intensity exercise programs in OA points to the need for research focusing on the minimal exercise intensity required for clinical effect as well as the highest intensity considered both safe and tolerable. In the same vein, Golightly et al. [58••] reviewed 39 studies focusing on the effects of aerobic and strength training exercise on OA-related pain and physical function. They showed that while both forms of exercise improved pain and function, there was no difference in effectiveness between the types of exercise programs studied (i.e., aerobic vs. strengthening regimens). Similarly, a recent meta-analysis of 48 randomized controlled trials on the effects of exercise on OA pain also showed an overall moderate benefit of exercise on pain, regardless of exercise type [59•]. However, Juhl et al. reported (i) that regular aerobic exercise (i.e., at least three times per week for 12 weeks) was more impactful than the intensity of aerobic exercise; (ii) that exercise programs using a single type of exercise (i.e., aerobic exercise, strength training) were more effective than programs mixing multiple types into the same exercise session [59•], but see [60]; and (iii) there was no evidence to support individualized exercise programs based on patient characteristics including radiographic severity of OA. In addition to these points, another important consideration is the duration of exercise-induced effects. A number of groups have indicated that exercise-induced OA benefits appear to fade once the exercise program is discontinued [56, 61, 62], underlining the importance of continued engagement in exercise programs in order to maintain beneficial effects. Indeed, improved adherence to exercise programs seems to be a stronger predictor of improvement in pain and physical function associated with knee OA than exercise frequency or intensity [63–65]. Taken together, while exercise appears to be beneficial for many types of chronic pain, a number of qualifications are in order: Firstly, no intensity or approach appears to be superior to another. Secondly, the available evidence does not appear to support individualized exercise programs. Thirdly, greater adherence to exercise programs yields better outcomes. Finally, benefits do not seem to last much longer than the duration of exercise program.

### Is Exercise Beneficial for Mental Health Impacts of Chronic Pain?

Aerobic exercise can improve depression to a level similar in scope to either psychological or pharmacological therapies [33]. Considering that depression affects between 20 and 35% of the chronic pain population [7, 66, 67] and can be considered a consequence of chronic pain [68, 69], establishing the effectiveness of exercise against comorbid depression has clear clinical relevance. While some studies indicate that aerobic exercise improved depression comorbid with fibromyalgia [70, 71], only 5 of the 21 Cochrane reviews assessed in Geneen et al. [53] reported mental health/depression outcomes, with positive yet somewhat variable results. While the existing literature indicates that exercise for depression comorbid with chronic pain is at least moderately effective, more randomized controlled trials of high methodological quality are needed.

### Mechanisms of Exercise-Induced Benefits in Chronic Pain

Activation of the endogenous opioid system has long been proposed to be the main biochemical mechanism underlying exercise-induced analgesia reviewed in [41, 65, 72, 73], as well as the euphoric state commonly referred to as ‘runners high’ [74]. However, these effects seem to occur acutely post-exercise and are largely dependent on exercise intensity, where a dose-response relationship exists between exercise intensity and reward/affective response [75] and perhaps also with anti-nociception [76]. Indeed, vigorous exercise (i.e., greater than 70% of the maximum aerobic capacity [VO_2_^max^], or the range required to improve cardiovascular fitness in healthy individuals) seems to be required to produce endogenous opioid release [77, 78], reviewed in [79] [80]. As such, benefits associated with lower intensity exercise (i.e., walking at an intensity substantially below 70% of VO_2_^max^) may involve longer-term engagement of the endogenous opioid system along with other endogenous systems. Another avenue by which exercise can reduce pain involves factors such as weight loss and other musculoskeletal benefits. Considering that excess weight plays an important structural role in OA pain [16, 81, 82], it is clear that weight loss can be beneficial [83, 84]. However, in terms of other musculoskeletal outcomes such as muscle function, the amount of benefit in pain does not necessarily correlate with the amount of benefit in these outcomes, suggesting that factors other than improved musculoskeletal function may be mediating pain relief [85]. Taken together, mechanisms of exercise-induced attenuation of persistent pain, especially pain relief associated with low-intensity exercise, remain unclear.

## Is Exercise Beneficial in Rodent Models of Chronic Pain?

**Fig. 1:**
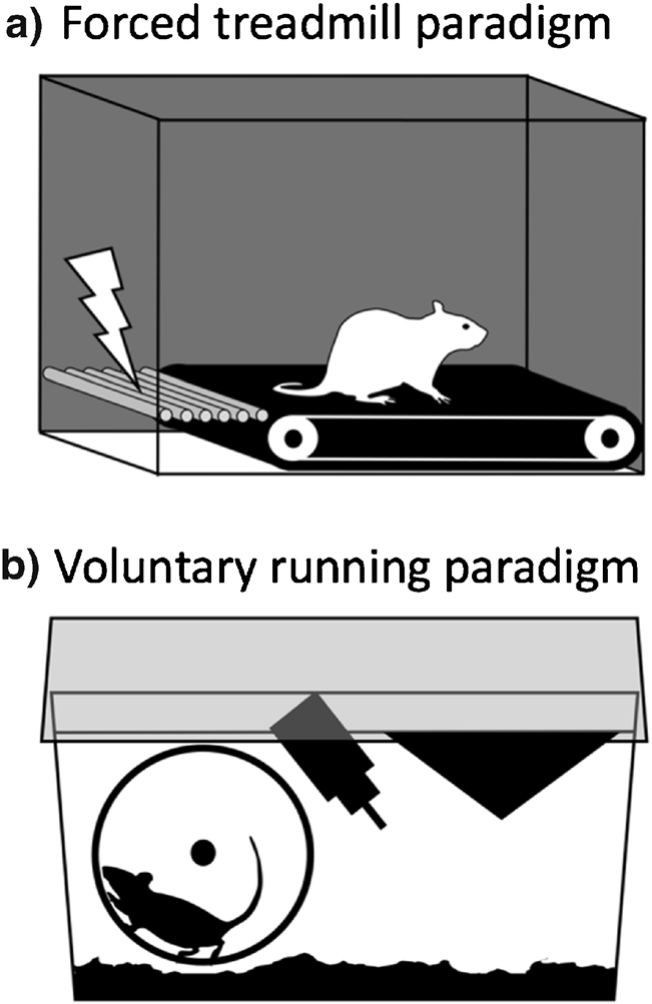
Rodent exercise paradigms. **a**. Forced treadmill running involves placing the rodent in an inescapable enclosure, either with or without an electrical shock grid to reinforce running behavior. Speed and incline of the treadmill can be adjusted. **b**. Voluntary wheel running paradigms yield either restricted or unrestricted access to the running wheel. During access, the rodent is free to run as much or as little as desired

Given the variability and other challenges inherent in human clinical trials, the apparent difficulty in isolating factors underlying exercise-induced analgesia is perhaps not surprising. Rodent studies, however, allow precise control of biological and environmental factors, as well as experimental interventions. As such, they should be well placed to probe these questions. A careful search of the biomedical research repository Pubmed (https://www.ncbi.nlm.nih.gov/pubmed) was performed using the search terms “exercise” and “chronic pain” or “pain” in non-human animal research. Only studies incorporating land-based physical exercise (i.e., voluntary wheel running or treadmill running; Fig. 1) as a primary intervention were included. Studies in which access to exercise was not ensured for each animal (i.e., environmental enrichment paradigms with a single voluntary exercise wheel for numerous cage mates) were not included in this review. Review articles, studies not focused on rodent models of persistent pain, and studies lacking pain/hypersensitivity outcomes were also excluded from this assessment. This search strategy revealed no less than 43 studies focused on the effects of exercise in rodent models of persistent pain (Tables 1 and 2). In the context of these studies, we discuss the impact of a number of factors including the type of chronic pain model (neuropathic, osteoarthritis, etc), species (rat, mouse), gender (male, female), exercise modality (forced treadmill running, voluntary wheel running), and exercise intensity characteristics (i.e., duration, frequency and velocity). While most studies used exercise as a therapeutic intervention (i.e., main exercise paradigm initiated post-injury), a number used exercise preventatively (i.e., exercise initiated pre-injury) or integrated both pre- and post-injury exercise into a single paradigm. Given that preventative paradigms may influence not only the maintenance of persistent pain states, but also the development of pain, we considered preventative exercise paradigms (Table 1) separately from therapeutic exercise paradigms (Table 2).

**Table 1:** Preventative exercise in rodent models of chronic pain

Reference	Pain model	Sex and Species	Exercise modality ± reinforcement	Pre-training	Exercise intensity	Maximal running velocity	Time delay between exercise and testing	Effective for mechanical hypersensitivity?	Effective for thermal hypersensitivity?	Other outcomes
Cobianchi et al. 2010	CCI (NP)	Male mice	Treadmill + shock	No	60 min/day, 5 days/week, 2 weeks	30 m/min	3 days	No	n/a	n/a
Stagg et al. 2011	SNL (NP)	Male rats	Treadmill + shock (some excluded for stress/not running)	No	10 min/day, 2 days/week, 2 weeks	18 m/min	~ 7 days	No	No	n/a
Sluka et al. 2013	Muscle	Male mice	Voluntary	No	24 h/day, 7 days/week, 5 days or 8 weeks	n/a	6 days	Yes: 8 weeks, effect lost by 1 week No: 5 days	n/a	Reduced phosphorylation of NMDA-NR1 in RVM
Gong et al. 2016	Incision	Female rats (neonatal)	Treadmill + shock (excluded poor runners)	No	30 min/day, 6 days/week, 3 weeks	15 m/min	4 days	No	No	n/a
Grace et al. 2016	CCI (NP)	Male rats	Voluntary	No	24 h/day 7 days/week, 6 weeks	n/a	1 day	Yes: effect lasted for 13 weeks post-exercise	n/a	Normalized IL-1b, GLT-1, P2X4R-BDNF. Reduced macrophage activity and CCL2 chemokine. Increased IL-10.
Kami et al. 2016A	PSNL (NP)	Male mice	Treadmill + prodding	No	10–60 min/day, 5 d/week, 2 weeks	7 m/min	2 days	No	No	n/a
Kami et al. 2016B	PSNL (NP)	Male mice	Treadmill + shock + prodding	No	10–60 min/day, 5 day/week, 2 weeks	7 m/min	2 dys	No	No	n/a
Leung et al. 2016	Muscle	Male and female mice	Voluntary	No	24 h/day 7 days/week, 8 weeks	n/a	6 days	Yes: Only tested 1 day post-injury. No gender differences.	n/a	Muscle macrophage and increasing IL-10
Sabharwal et al. 2016	Muscle	Male and female mice	Voluntary	No	24 h/day 7 days/week, 5 days or 8 weeks	n/a	6 days	Yes: 5 days = 8weeks. Only tested 1 day post-injury. No gender differences.	n/a	Prevented development of autonomic dysfunction (i.e., reduced HRV)
Wakaizumi et al. 2016	PSNL (NP)	Male mice	Treadmill	No	60 min/day, 5 days/week, 2 weeks	6–12 m/min	5 days	No	n/a	n/a
Safakhah et al. 2017	CCI (NP)	Male rats	Treadmill	5 days, 10 min/day, 10 m/min	30 min/day, 5 days/week, 3 weeks	16 m/min	27 days	No	No	n/a

*CCI* chronic constriction injury, *PSNL* partial sciatic nerve ligation, *SNL* spinal nerve ligation, *Muscle* muscle pain, *Incision* incision, *NP* neuropathic pain model, *n/a* not applicable, *PID* post-injury day, *m/min* meters per minute

**Table 2:** Therapeutic exercise in rodent models of chronic pain

Reference	Pain model	Sex and species	Exercise modality ± reinforcement	Pre-training	Exercise initiated	Exercise intensity	Maximal running velocity	Effective for mechanical hypersensitivity?	Effective for thermal hypersensitivity?	Other outcomes
Hutchison et al. 2004	SCI (NP)	Female rats	Treadmill + positive reinforcement	1 week, 12 m/min	PID 4	25 min/day, 5 days/week, 7 weeks	12 m/min	Yes	n/a	Restored BDNF signaling
Bement et al. 2005	Muscle	Male rats	Treadmill (some excluded)	3 days, 5 min/day, 3 m/min	PID 1	30 min/day, 5 days	6 m/min	Yes	n/a	Opioid-dependent effect of exercise
Cobianchi et al. 2010	CCI (NP)	Male mice	Treadmill + shock (some excluded)	2 weeks, 60 min/day, 5 days/week, 30 m/min	PID 3	60 min/day, 5 days, or 8 weeks	30 m/min	Yes 5 days No > 5 days	n/a	Shorter duration reduced microglia/astrocyte expression, better nerve regeneration
Korb et al. 2010	SNSR (NP)	Male rats	Treadmill	4 days, 10 min/day, 5 m/min, then max test	PID 7	60 min/day, 5 days/week, 4 weeks	9 m/min	Yes	n/a	Serotonin activity in the spinal cord
Sharma et al. 2010	Muscle	Female mice	Treadmill + prodding	3 days, 13 m/min	PID 5	45 min/day, 5 days/week, 3 weeks	16 m/min	Yes	n/a	NT-3 expression increased
Stagg et al. 2011	SNL (NP)	Male rats	Treadmill + shock (some excluded for stress/not running)	2 weeks, 2 day/week, 10 min/day, 18 m/min, 8% grade	PID 7 or 28	30 min/day, 3 days/week or 5 days/week, 5 weeks	10–16 m/min	Yes: 3 days/week = 5 days/week Yes: 7 days delay = 8 days delay. Yes: 16 m/min. No: 10 m/min In all cases, exercise effects lost ~ 5 days.	Yes	Opioid-mediated effects in the RVM and the PAG
Shankarappa et al. 2011	Diab (NP)	Male rats	Treadmill + shock	5 days, 60 min/day, 18 m/min	n/a	60 min/day, 5 days/week, 10 weeks	18 m/min	Yes	No change in diabetic rats	Opioid-mediated. Ca2+-mediated changes in DRG neurons
Chen et al. 2012	CCI (NP)	Male rats	Treadmill (no shock)	3 days, 15 min/day, 20 m/min	PID 2	60 min/day, 5 days/week, 6 weeks	30 m/min	Yes	Yes	Increased Hsp-72 and decreased IL-1b, TNF⍺
Chen et al. 2013A	Diab (NP)	Male rats	Treadmill (some excluded)	n/a	Not indicated	60 min/days, 7 days/week, 8 weeks	20 m/min	Yes	Yes	Reduced blood glucose levels and increased Hsp-72 but no effect on IL-6, TNF⍺
Chen et al. 2013B	Incision	Male rats	Treadmill	3 days 15 min	PID 8	55 min/day, 5 days/week, 4 weeks	18 m/min	Yes, partial reversal	n/a	Lower NMDA-NR1, TNF⍺, and IL-6 in the spinal cord
Cobianchi et al. 2013	SNSR (NP)	Female rats	Treadmill + shock	n/a	PID 3	60 min/day, 5 days	19 m/min	Yes, partial reversal	No	Improved axonal regeneration, reduced BDNF, NGF, and GDNF in DRG
Groover et al. 2013	Diab (NP)	Male mice	Voluntary	n/a	PID 1	24 h/day 7 days/week, 12 weeks	n/a	Yes: 6wks to take effect.	No change in diabetic rats	Reduced metabolic abnormalities. Improved innervation and neurotrophin levels (TrkA, NGF, BDNF)
Morimoto et al. 2013	Cast	Male rats	Treadmill	3 days	PID 3	30 min/day, 3 days/week, 2 weeks	12 m/min	Yes	n/a	Range of motion and calf muscle atrophy improved
Chen et al. 2014	Incision	Male rats	Treadmill + shock	n/a	PID 6	60 min/day, 5 days/week, 4 weeks	18 m/min	Yes, partial reversal	n/a	Reduced substance P, IL-1b, IL-6, in DRG
Detloff et al. 2014	SCI (NP)	Female rats	Automated running wheels (forced)	n/a	PID 5	20 min/day, 5 days/week, 5 weeks	14 m/min	Yes: Immediately effective.	No	GDNF, artemin maintained at normal levels
Bobinski et al. 2015	SNC (NP)	Male mice	Treadmill	1 week, 10 min/day, 10 m/min	PID 3	30 min/day, 5 days/week, 2 weeks	10 m/min	Yes	n/a	Brainstem serotonin increased, while TNF⍺ and IL-1b were reduced
Chen et al. 2015	Diab (NP)	Male rats	Treadmill + prodding	3 days, 15 min/day, 10 m/min	PID 3	60 min/day, 7 days/week, 4 weeks	25 m/min	Yes	Yes	Increased IL-10 and decreased IL-6, TNF⍺, MDA
Chuganji et al. 2015	Cast	Male rats	Treadmill	n/a	PID 0	30 min/day, 5 days/week, 8 weeks	15 m/min	Yes	n/a	β-endorphin increased in PAG and hypothalamus
Lopez-Alvarez et al. 2015	SNSR (NP)	Female rats	Treadmill	1 h, 19 m/min	PID 3	60 min/day, 5 days or 10 days	19 m/min	Yes	Yes	Prevented changes in peripheral innervation and disruption of Cl co-trans. Reduced NGF, BDNF
Kim et al. 2015	CCI (NP)	Male rats	Treadmill	n/a	Not indicated	30 min/day, 5 days/week, 4 weeks	22 m/min	Yes	Yes, partial reversal	Reduced Mu opioid receptor expression in RVM and spinal cord
Luan et al. 2015	Disc	Male rats	Treadmill	n/a	PID 14	40 min/day, 7 day/week, 1–8 weeks	13 m/min	Yes	n/a	Improved degenerated discs and neurogenesis in and around disc
Sheahan et al. 2015	SNI (NP)	Male mice	Voluntary	n/a	PID 8–10	2–12 h/day, 5–6 d/week, 1 or 4 weeks	n/a	No	No	Ineffective in reversing muscle wasting or denervation.
Yoon et al. 2015	Diab (NP)	Male rats	Treadmill	2 days, 20 min/day, 5 m/min	PID 7	60 min/day, 5 days/week, 6 weeks	10 m/min	Yes	Yes	Increased enkephalin, HSP70; decreased TNF⍺, IL-1b, TRPV1, TRPM8, pp38 in the DRG
Detloff et al. 2016	SCI (NP)	Female rats	Automated running wheels (forced)	n/a	PID 14 or 28	20 min/day, 5 days/week, 5 weeks	14 m/min	No	n/a	
Gong et al. 2016	Incision	Female rats (neonatal)	Treadmill + shock (excluded poor runners)	n/a	PID 22	30 min/day, 6 days/week, 2 weeks	15 m/min	No	No	Reduced high P38 MAPK, 1L-1b, TNF⍺ (but not IL-6).
Grace et al. 2016	CCI (NP)	Male rats	Voluntary	n/a	PID 0 or 14	24 h/d 7 days/week, 2 weeks or 11 weeks	n/a	Yes: 2wks = 11wks	n/a	Normalized IL-1b, GLT-1, P2X4R-BDNF. Reduced macrophage activity and CCL2 chemokine. Increased IL-10.
Kami et al. 2016A	PSNL (NP)	Male mice	Treadmill + prodding	10–60 min/day, 7 m/min, 2 weeks	PID 2	60 min/day, 5 day/week, 5 days	7 m/min	Yes	Yes	Blocked decrease in GABA/GAD65/67 reductions in dorsal horn
Kami et al. 2016B	PSNL (NP)	Male mice	Treadmill + prodding	10–60 min/day, 7 m/min, 2 weeks	PID 2	60 min/day, 5 day/week, 5 day	7 m/min	Yes	Yes	Reduced histone deacetylase1+/CD11b+ microglia in spinal cord
Wakaizumi et al. 2016	PSNL (NP)	Male Mice	Treadmill	3 options: - None - 2 weeks, 1 h/day, 5 days/week, 6 m/min - 2 weeks, 1 h/day, 5 days/week, 12 m/min	n/a	60 min/day, 5 days/week, 1 week (fast) or 2 weeks (slow)	6 m/min or 12 m/min	Yes: 2wks pre + 2wks post, slow. Yes: 2wks post, slow. Yes: 2wks pre + 1wk post, fast. No: 1wk post, fast.	Yes: 2wks, slow	Mesolimbic dopamine activity
Allen et al. 2017	MIA (OA)	Male Rats	Treadmill + shock	n/a	PID 10	30 min/day, 4 days/week, 4 weeks	16 m/min	Yes	n/a	Opioid-mediated effects. Exercise improved trabecular bone microarchitecture.
Arbat-Plana et al. 2017	SNSR (NP)	Female Rats	Treadmill + shock	n/a	PID 3	60 min/day, 5 days/week, 2 weeks	18 m/min	Yes	n/a	BDNF/TrkB signaling maintaining functional spinal neuro-circuitry.
Bobinski et al. 2017	SNC (NP)	Male Mice	Treadmill	n/a	PID 3	30 min/day, 5 days/week, 2 weeks	10 m/min	Yes	n/a	Increased IL-4
Cormier et al. 2017	MIA (OA)	Male Rats	Voluntary	1 or 3 weeks	PID 0	24 h/day 7 days/week, 3 weeks	n/a	Yes: 3wks pre + 3wks post. No: 1wk pre + 3wks post.	n/a	Improved trabecular bone microarchitecture.
Huang et al. 2017	CCI (NP)	Male rats	Treadmill + prodding	n/a	PID 8	30 min/day, 7 days/week, 3 weeks	16 m/min (8% grade)	Yes	Yes	Reduced IL-6 and TNF⍺ and increased IL-10
Lopez-Alvarez et al. 2017	SNSR (NP)	Female rats	Treadmill	60 min, 19 m/min	PID 3	60 min/day, 12 day	19 m/min	Yes	Yes	Monoaminergic (5HT) descending pathways, and BDNF and microglia in locus coeruleus
Pitcher et al. 2017	CFA (OA)	Male rats	Voluntary	n/a	PID 3	2 h/day, 4 d/week, 3 weeks	n/a	Yes	Yes	Decreased stress, increased HRV, no change in swelling. No association between running and benefit
Safakhah et al. 2017	CCI (NP)	Male rats	Treadmill (some excluded)	5 days, 10 min/day, 10 m/min	PID 5	30 min/day, 5 days/week, 3 weeks	16 m/min	Yes	Yes	Decreased TNF⍺ and increased FRAP
Tsai et al. 2017	CCI (NP)	Male rats	Treadmill + prodding	n/a	PID 6	30 min/day, 7 days/week, 3 weeks	14–16 m/min (8% grade)	Yes: 8% incline better than 0%.	Yes: 8% incline better than 0%.	Decreased TNF⍺ and IL-6 and prevented downreg of IL-10
Whitehead et al. 2017	CCI (NP)	Male rats	Voluntary	1wk, 60 min/d	PID 2–3	60 min/day, 7 days, 18 days	n/a	No	n/a	
Yamaoka et al. 2017	SNL (NP)	Female rats	Treadmill	n/a	PID 1	20 min/day, 5 days/week, 6 weeks	20 m/min (10° incline)	Yes	Yes, partial reversal	

*CCI* chronic constriction injury, *PSNL* partial sciatic nerve ligation, *SNL* spinal nerve ligation, *SNI* spared nerve injury, *SNSR* sciatic nerve section and repair, *SCI* spinal cord contusion injury, *SNC* sciatic nerve crush, *Diab* diabetic neuropathy, *MIA* Mono-iodoacetate, *CFA* complete Freund’s adjuvant, *Muscle* muscle pain, *Incision* incision, *Cast* cast immobilization, *Disc* disc degeneration, *OA* osteoarthritis model, *NP* neuropathic pain model, *n/a* not applicable, *PID* post-injury day, *m/min* meters per minute

### Preventative Exercise

A number of studies incorporated experiments that were designed specifically to assess the effectiveness of preventative exercise on the development of persistent pain in rodents (Table 1). Of the 11 studies employing preventative exercise paradigms, 7 used neuropathic pain models (64%), 3 used a model of chronic muscle pain (27%), and 1 study used a model of incisional pain (9%). A total of eight studies used male rodents (73%), whereas two studies used both males and females (18%) and one used only females (9%). Seven studies used mice (64%), while the remaining four used rats (36%). Seven studies used a forced treadmill running paradigm (64%), while the remaining four employed voluntary wheel running (36%). In terms of exercise characteristics, all of the studies employing voluntary running allowed rodents unrestricted access to running wheels for a duration between 5 days and 8 weeks. While the duration of forced treadmill running studies was fairly uniform (i.e., 2–3 weeks), their frequency ranged from 10 min per day twice a week to 1 h per day for 5 days a week. Maximal running velocity in studies using voluntary running wheels was not reported. However, the maximal running velocity used in forced treadmill studies ranged between 6 and 30 m/min.

A total of four studies reported that preventative exercise paradigms reduced mechanical hypersensitivity [86–88, 89••]. These studies all used voluntary exercise, where rodents had unrestricted access to running wheels. Moreover, three of the four voluntary exercise studies employed a muscle pain model [86, 88, 89••], whereas the fourth used a neuropathic pain model [87]. Exercise of longer duration, beginning 6–8 weeks prior to pain induction, appeared to be most effective [86–88] but see [89••]. In terms of the duration of exercise-induced benefits, while Grace et al. reported long-lasting effects of preventative exercise for neuropathic pain [87], the beneficial effects of exercise on chronic muscle pain lasted no longer than 3 days post-induction [86, 88, 89••]. In the two studies using both male and female rodents, no sex differences in the effects of exercise were observed [88, 89••]. Of the five studies assessing thermal hypersensitivity, all employed forced treadmill running paradigms. However, none found preventative exercise to be effective at reducing thermal hypersensitivity (Table 1). Overall, among the studies where preventative exercise was not effective at reducing hypersensitivity, all used forced treadmill running in models of neuropathic pain [90, 91•, 92–96]. As such, it appears that the exercise paradigm (i.e., voluntary exercise), the amount of running (i.e., unrestricted wheel access), and possibly the model of chronic pain, may all contribute to the effectiveness of preventative exercise paradigms in rodent models of chronic pain.

### Therapeutic Exercise

A total of 40 studies initiated the main exercise program therapeutically, after the onset of experimental models of chronic pain in rodents (Table 2). Over half of these studies used therapeutic exercise that was preceded by some level of pre-training prior to injury (21/40, or 52.5%). None of these studies reported an analgesic effect of pre-training on early post-injury hypersensitivity. As such, these studies are considered in the context of therapeutic exercise.

Of the 40 studies employing therapeutic exercise paradigms, 29 used neuropathic pain models (72.5%), 3 used models of osteoarthritis (7.5%), 3 used incision models (7.5%), and 5 used other models (12.5%). A total of 30 studies used male rodents (75%), whereas 10 studies used females (25%). The majority of studies used rats (31 or 77.5%), while the remaining nine studies used mice (22.5%). In most cases, a forced running paradigm was used (34 studies, or 85%). In these studies, exercise was performed for between 20 and 60 min/day for 4–7 days/week. The six studies (15%) using voluntary wheel running generally allowed longer wheel access (2–24 h/day [87, 97, 98, 99••, 100••] but see [101]) for 4–7 days per week. However, it should be noted that the actual running time in voluntary exercise paradigms is unknown because rodents do not necessarily engage in constant running during periods of wheel access. The duration of the voluntary running paradigms ranged between 1 and 12 weeks, but most lasted between 2 and 4 weeks. Furthermore, only one study using voluntary exercise reported the average running velocity: Pitcher et al. [100••] showed that the average velocity of both sham and OA groups was approximately 45 m/min, which is comparable to other studies using voluntary wheel running [102•, 103]. On the other hand, the maximal velocity at which rodents were forced to run on treadmills ranged between 6 and 30 m/min. While it is certainly possible to train rodents to run on a treadmill at velocities of 30 m/min, maintaining this velocity for more than a few minutes appears to require substantial aerobic pre-training (8–10 weeks) at oxygen intake levels approaching maximal capacity (i.e., VO^2^_max_) [104]. In fact, some groups report difficulty in forcing rats to run faster than 20 m/min [102•], and approximately 25% of mice forced to run at 12 m/min on treadmills cease running by 10–15 min, and around 50% cease running by 20–25 min [105]. Considering that the forced running studies discussed here generally employed little or no pre-training prior to relatively high exercise intensities, it is somewhat surprising that rodents with painful hind paw injuries were able to complete the studies. Indeed, a number of studies reported that some rodents were excluded due to stress or refusal to run [90, 91•, 92, 96, 106, 107], while another reported that some mice discontinued running at the 12 m/min velocity during the therapeutic exercise phase [95]. Moreover, a number of studies indicated that electric shocks or physical encouragements were required to ensure continued running [90, 91•, 92–94, 108^•^, 109–113, 114••, 115–117]. Under these conditions, the vast majority of studies (36 or 90%) reported that at least some form of therapeutic exercise was effective in reducing or reversing mechanical hypersensitivity. While three of the four studies showing no beneficial effect of therapeutic exercise on mechanical hypersensitivity initiated exercise 8 or more days after injury [92, 98, 118], other studies using similar delays in initiation of exercise were effective [87, 91•, 114••, 116, 119, 120]. Similarly, while two of the four studies showing no beneficial effect of therapeutic exercise on mechanical hypersensitivity used voluntary exercise paradigms [98, 101], other studies using voluntary exercise were effective [87, 97, 99••, 100••]. Of the 36 studies showing that exercise was effective, a few indicate that some approaches were not as effective as others [91•, 95, 99••]. Specifically, Stagg et al. reported that 3 weeks at a slower treadmill speed of 10 m/min did not improve mechanical hypersensitivity, whereas the same duration at a higher speed of 16 m/min was effective [91•]. On the other hand, Wakaizumi et al. showed that while 2 weeks of slow running (6 m/min) was effective, 1 week of faster running (12 m/min) was not effective [95]. Finally, Cormier et al. indicated that while 3 weeks of pre-training followed by 3 weeks of therapeutic running resulted in beneficial effects, 1 week of pre-training followed by 3 weeks of therapeutic running was not beneficial [99••]. Importantly, in all of these cases, other studies using similar exercise parameters show effectiveness. Consequently, no particular factors appear to be consistently associated with either effectiveness or ineffectiveness of therapeutic exercise.

A total of 22 studies incorporated measures of thermal hypersensitivity (Table 2). Two of these studies, both using models of diabetes-induced pain, did not observe thermal hypersensitivity in diabetic rodents [97, 110], which is in contrast to other studies using diabetic models [107, 113, 121]. Of the remaining 20 studies, 16 showed that exercise was effective in reducing or reversing thermal hypersensitivity (80%). Of the four studies in which no effect of exercise was observed, three were conducted in female rats [92, 111, 122]. Nonetheless, three other studies also using female rats showed improved thermal hypersensitivity [123–125]. Similarly, while three of the four studies in which no effect of exercise was observed utilized neuropathic pain models [98, 111, 122], a number of other studies successfully employed therapeutic exercise against neuropathic pain-induced thermal hypersensitivity [123–125]. Therefore, among the studies considered here, therapeutic exercise appears to be an effective method of reducing thermal hypersensitivity. However, no specific factors appear to be related to exercise-induced improvements in thermal hypersensitivity.

### Mechanisms of Exercise-Induced Benefits in Rodent Models of Chronic Pain

As illustrated in Tables 1 and 2, both voluntary wheel running and forced treadmill running promote favorable outcomes in a number of physiological systems impacted by persistent pain. Exercise improves measures of neurological function in the periphery and spinal cord [90, 94, 97, 111, 115, 119, 121, 123] as well as improved musculoskeletal outcomes [99••, 114••, 120, 126]. In addition, exercise improved neurotrophic receptor signaling in the spinal cord and periphery [87, 97, 108•, 109, 111, 115, 122–124]; restoration to pre-injury levels of cytokines and other neuroimmune products in the brainstem, spinal cord, and periphery [87, 88, 90, 93, 96, 112, 113, 116, 117, 119, 121, 123, 124, 127, 128]; and increased endogenous opioid activity in the rostroventral medulla (RVM), spinal cord, and dorsal root ganglia (DRG) [91•, 106, 110, 114••, 121, 129, 130]. Importantly, while endogenous opioid-mediated mechanisms can produce analgesia at acute post-exercise time points, some studies suggest that longer-term endogenous opioid-mediated effects also occur. Specifically, Stagg et al. [91•] and Allen et al. [114••] demonstrated that naloxone blocks exercise-induced analgesia even when injected at time points far beyond the potential acute effects of exercise. As such, regular exercise may induce long-term tonic changes in endogenous opioid tone that promote analgesia.

### Does Exercise Alter Stress in Rodent Models of Chronic Pain?

Of the 43 studies discussed here, only two incorporated stress measures [89••, 100••], and both employed voluntary wheel running paradigms. Sabharwal et al. demonstrated that as little as 5 days and up to 8 weeks of unrestricted access to running wheels prior to injury prevented injury-induced reductions in heart rate variability (HRV), a measure of autonomic health known to be negatively impacted by stress [131–134]^,^ reviewed in [135] and chronic pain [136]. In the same vein, Pitcher et al. showed that following induction of a rat model of OA, 3 weeks of modest access to running wheels (2 h/day, 4 days/week) improved both HRV and plasma levels of the stress hormone corticosterone. Pitcher et al. also assessed the relationship between exercise intensity and pain and stress outcomes [100••]. Similar to studies in humans, exercise intensity (i.e., total distance, average velocity) was unrelated to the degree of benefit in both pain and stress, a finding that challenges the widely held belief that more exercise yields better outcomes. Others have shown a similar lack of accord between the intensity of voluntary running and measures of stress/reward [137••, 138–142]. Overall, relatively low levels of self-regulated exercise appear to be protective against persistent pain and persistent pain-induced stress.

### Conclusions

The vast majority of rodent studies discussed here report beneficial effects of exercise in models of chronic pain. These benefits were accrued from both voluntary and forced exercise paradigms incorporating a diversity of exercise characteristics. To some extent, exercise-induced improvements in rodent models of chronic pain mirror the conclusions expressed in human chronic pain meta-analyses, where no intensity or approach appears to be superior to another, and exercise benefits do not seem to be related to any particular facet of either the type of injury or the exercise paradigm. In fact, there appears to be only one consistent factor among rodent studies showing exercise efficacy: regular exercise. In each study, rodents are exposed to exercise on a regular basis for a period of time. Similarly, regular exercise seems to be among the most important factors underlying exercise-induced benefits in the human literature. That said, this brief review of a relatively limited body of rodent literature was not meant to be meta-analytic (i.e., incorporating comparable data from multiple sources for statistical re-analysis). As such, it is possible that it was not sufficiently refined to detect an existing relationship between certain exercise characteristics and analgesic outcomes.

How might regular exercise exert its beneficial effects independently of other exercise-related factors? A number of rodent studies suggest that exercise-induced modulation of the immune/inflammatory response may play a role [87, 88, 90, 93, 96, 112, 113, 116, 117, 119, 121, 123, 124, 127, 128]. Indeed, stress-related changes in immune reactivity has been proposed as a major contributing factor in the development and maintenance of chronic pain [143]. Exercise, particularly regular aerobic exercise, appears well placed to reduce the impact of an altered immune/inflammatory responses in chronic diseases such as diabetes and obesity [144–146]. As such, it may be similarly effective in chronic pain states. Perhaps even more prominent than the immune response, the human literature emphasizes a role for endogenous opioids in exercise-induced analgesia reviewed in [41, 65, 72, 73]. However, the apparent intensity-dependence of acute exercise for activation of the endogenous opioid system [75–78]^,^ reviewed in [79, 80] seems to argue against its involvement in low-intensity exercises such as walking. In the rodent exercise literature, activity of the endogenous opioid system is also widely reported [91•, 106, 110, 114••, 121, 129, 130]. However, these studies employed forced treadmill running, which can be highly stressful [102•, 147–154]. Indeed, forced running is more stressful than voluntary exercise when both paradigms are compared directly [102•, 155, 156]. Stressors involving unpleasant, inescapable contexts (i.e., cold-water swim, restraint) and/or electric shock are commonly used to evoke stress-induced analgesia (SIA), a stress-induced reduction in pain sensitivity related to increased endogenous opioid or cannabinoid activity [157, 158]. By definition, the forced running paradigm is inescapable and often incorporates electric shock plates or other negative reinforcements to promote running (Fig. 1), and forced treadmill walking has been used by at least one research group as a model of stress-induced analgesia [159–162]. Of the 43 studies considered here, almost 80% employed forced running paradigms, the vast majority of which reported exercise-induced analgesia (Tables 1 and 2). Of these, 41% indicated that negative reinforcements, in the form of electric shock or physical stimuli such as manual prodding of the rodent, were used to promote running. Only one study stated explicitly that electrical shock was *not* used to reinforce running behavior [128], while another indicated that positive reinforcement (i.e., sweetened water) was used to reinforce treadmill running [108•]. The remaining forced running studies did not state whether or not negative reinforcement was used. Considering the clear potential for stress-induced analgesia in forced running paradigms, it is surprising that the only two rodent studies including stress outcomes both employed voluntary running paradigms [89••, 100••]. On the other hand, at least one study has reported that relatively intense forced treadmill running does not increase tail flick latencies, a common assay for stress-induced analgesia in rodents [163]. In addition, forced running paradigms can activate reward centers in the rodent brain if the animals are pre-trained appropriately [164], as well as produce beneficial physiological effects in some contexts [153, 165–167]. Nevertheless, given the absence of stress measures in the forced running studies, it is not possible to exclude the potential contribution of stress-induced analgesic effects in their results.

While voluntary running paradigms may avoid the influence of stress-induced analgesia, it is well known that rodents will often exhibit very high levels of running behavior when given unrestricted access to running wheels, where rats have been reported to attain peak velocities of approximately 160 m/min and mice up to 210 m/min for very brief bursts [168, 169]. As aforementioned, high-intensity activity triggers the endogenous opioid system [77, 78]^,^ reviewed in [79] [80], and voluntary wheel running can certainly increase endogenous opioid levels [138, 139, 170–174]. However, such high-intensity physical activity cannot be said to represent a clinically relevant therapeutic approach for most chronic pain patients. On the other hand, evidence in rodents suggests that regular exercise may also enhance tonic activity of the opioid system beyond acute post-exercise time points [91•] [114••]. As such, a better understanding of the effects of long-term exercise on tonic endogenous opioid activity is needed. Only three rodent studies assessed the effect of more modest levels of voluntary exercise [98, 100••, 101]. Of these, only one demonstrated that exercise was effective [100••], indicating that additional research directly assessing the role of stress in the analgesic effects of running is required, incorporating experimental paradigms that more accurately represent the human chronic pain population. Taken together, the human and rodent literature suggest that regular exercise, even at modest levels, can be beneficial for chronic pain. However, the current state of the literature precludes a nuanced understanding of optimal exercise parameters and putative biological mechanisms.
